# Changes in vegetation phenology on the Mongolian Plateau and their climatic determinants

**DOI:** 10.1371/journal.pone.0190313

**Published:** 2017-12-21

**Authors:** Lijuan Miao, Daniel Müller, Xuefeng Cui, Meihong Ma

**Affiliations:** 1 School of Geography, Nanjing University of Information Science and Technology, Nanjing, China; 2 Leibniz Institute of Agricultural Development in Transition Economies, Halle (Saale), Germany; 3 Geography Department, Humboldt-Universität zu Berlin, Berlin, Germany; 4 School of Mathematics and Statistics, University College Dublin, Dublin, Ireland; 5 College of System Science, Beijing Normal University, Beijing, China; 6 College of Water Science, Beijing Normal University, Beijing, China; INRA - University of Bordeaux, FRANCE

## Abstract

Climate change affects the timing of phenological events, such as the start, end, and length of the growing season of vegetation. A better understanding of how the phenology responded to climatic determinants is important in order to better anticipate future climate-ecosystem interactions. We examined the changes of three phenological events for the Mongolian Plateau and their climatic determinants. To do so, we derived three phenological metrics from remotely sensed vegetation indices and associated these with climate data for the period of 1982 to 2011. The results suggested that the start of the growing season advanced by 0.10 days yr-1, the end was delayed by 0.11 days yr-1, and the length of the growing season expanded by 6.3 days during the period from 1982 to 2011. The delayed end and extended length of the growing season were observed consistently in grassland, forest, and shrubland, while the earlier start was only observed in grassland. Partial correlation analysis between the phenological events and the climate variables revealed that higher temperature was associated with an earlier start of the growing season, and both temperature and precipitation contributed to the later ending. Overall, our findings suggest that climate change will substantially alter the vegetation phenology in the grasslands of the Mongolian Plateau, and likely also in biomes with similar environmental conditions, such as other semi-arid steppe regions.

## Introduction

The Intergovernmental Panel on Climate Change (IPCC) suggested an increase in land surface and ocean temperature of approximately 0.85°C from 1880 to 2012 in its Fifth Assessment Report [1]. Climate change will impact ecosystems in different ways, including by altering plant phenology [2]. Phenology is a vital indicator of ecosystem dynamics because it affects carbon and nutrient cycles of plant growth and thus food production [2, 3]. Numerous studies have documented an earlier date of spring phenology and a later date of the autumn phenology at both field and regional scales [4–6]. These changes were linked to climatic variations and especially to increasing temperature and higher precipitation [7, 8]. For example, responses of the phenology to climate change have been explored in United States [9, 10], Europe [11–13], Africa [14, 15], and parts of China [16, 17]. However, evidence of plant phenology and its relations with climatic variables mainly relies on *in situ* records that provide accurate phenological information at the species level but are limited in their spatial scope [16].

A region that has thus far received little attention in phenological studies is the Mongolian Plateau that covers parts of Northeastern China and the entirety of Mongolia. This is unfortunate because the vegetation in this region is highly vulnerable to climate change and climate extremes [18]. Moreover, the Mongolian Plateau is characterized by a common steppe environment and by important cultural, economic, and ecological interconnections that transcend political boundaries [19]. Phenological shifts are of crucial importance in this arid and semi-arid region, particularly for the millions of nomadic pastoralists who rely on grazing of the grassland biomass [16, 17]. Several studies investigated the impacts of climate change on vegetation cover of the Mongolian Plateau, including assessing effects of the vegetation change on the livestock herders [19, 20]. However, the influences of climate variables on phenological dynamics are elusive for the arid and semi-arid areas in general, and for the Mongolian Plateau in particular.

Remote sensing has been frequently used to measure phenology and changes in phenological events for large areas, and the most common tool has been to calculate the normalized difference vegetation index (NDVI) from satellite data [21, 22]. Several phenological metrics can be derived from time series of remote sensing data, including the start of the growing season (SOS) and the end of the growing season (EOS). These methods differ in the interpretation of phenological events that are extracted from the NDVI seasonal cycle [21–24].

The majority of studies about Eastern Asia have focused on the province of Inner Mongolia in China but used only short time series. For example, Sha et al. [25] employed NDVI time series from SPOT-VGT and found that the steppe desert in Inner Mongolia exhibited an earlier SOS and later EOS from 1998 to 2012. Similarly, Gong [26] calculated an advancement of SOS by 5.79 days and a delay of EOS by 5.07 days during 2002 to 2014. However, few studies have addressed the phenological dynamics in the neighboring country of Mongolia, which is unfortunate given the country’s huge grassland resources that have long been shaped by the nomadic herding cultures. Moreover, comparing the phenological dynamics between Mongolia and the Chinese province of Inner Mongolia can reveal interesting insights into the effects of the vegetation response to the different development pathways between the two cases.

In this study, we measured the phenological dynamics across the entire Mongolian Plateau using remotely sensed NDVI and quantified the climatic determinants of these changes. More specifically, we investigated the following two questions: How did plant phenology change across the region from 1982 to 2011 among different land-cover categories? How did different climatic parameters affect the observed phenological dynamics?

## Materials and methods

### Study area

**Fig 1:**
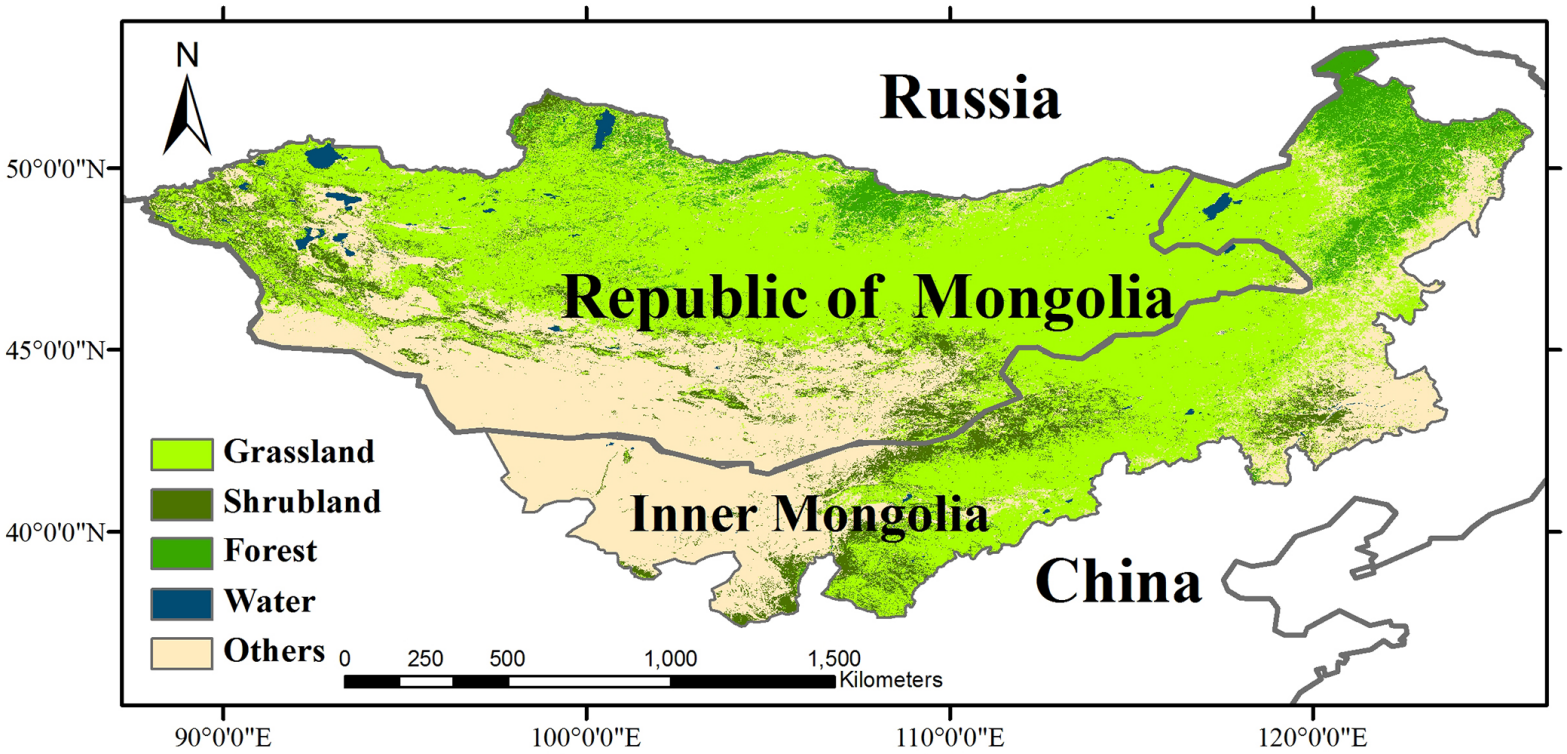
Land-cover map of the Mongolian Plateau (source: MODIS land cover product 2004).

We explore the phenological shifts over the Mongolian Plateau that extends over 2.6 million km^2^ across much of Inner Asia (Fig 1). The region contains one of largest grassland areas in the world. The grasslands have been used by nomadic herdsmen for centuries and provide fodder for widespread non-nomadic animal husbandry [19]. The region has an average elevation of 1,580 m and the climate is characterized by a temperature range from -45°C to +35°C. Annual precipitation averages 200 mm, with large differences across the region and over time [27]. Water is a scarce resource in the study area due to the low precipitation coupled with high evapotranspiration during the hot summer season. Moreover, rainfall is highly variable [28]. The Mongolian Plateau has also experienced the shrinkage of lake volumes during recent decades due to intensive human extraction that has been exacerbated by climate changes [29].

### Data

#### Land cover

We used the MODIS land cover map from 2004 at 0.05 degrees (https://modis.gsfc.nasa.gov/data/dataprod/mod12.php) to explore the phenological features among different land-cover categories. We grouped the 17 land cover types that occurred in the Mongolian Plateau into grassland (grassland and savanna), shrubland (closed and open shrubland), forest (evergreen needleleaf forest, deciduous needleleaf forest, deciduous broadleaf forest and mixed forest), and “other” with all other land cover categories (mainly barren or sparsely vegetated, wetlands and built up) (Fig 1). The predominant land cover type is grassland, which covers nearly 60% of the region.

#### NDVI

We used NDVI3g (third generation) data from the Global Inventory Modeling and Mapping Studies (GIMMS) available at https://nex.nasa.gov/nex/projects/1349/. These data were collected from the Advanced Very High Resolution Radiometer (AVHRR) sensors aboard a family of satellites from the National Oceanic and Atmospheric Administration (NOAA). The NDVI measurements from AVHRR that are available via GIMMS consist of 15-day composites and constitute the longest series of global NDVI from 1982 to 2011 with a spatial resolution of approximately 8 km [30]. The GIMMS data are corrected for changes in satellite sensors over time, unstable atmosphere conditions, and noise from non-vegetated areas [31–33]. For these reasons, the data have been widely applied in ecosystems research [27, 34–36].

#### Climate

We relied on monthly temperature, precipitation, and insolation (i.e., the sum of received solar radiation) from the Climate Research Unit—National Centers for Environmental Prediction (CRU-NCEP) v5 data. These data are hosted by the Climate Research Unit at the University of East Anglia. The data include the CRU-TS v3.2 with 0.5° x 0.5° monthly resolution from 1901 to 2009 and data from the NCEP reanalysis project at 2.5° x 2.5° spatial resolution and 6-hour time steps since 1948 [37]. The observed changes in the preseason climatic variables are summarized for the study area in S1 Fig.

### Phenology extraction and further analysis

While seasonal NDVI dynamics are typically a good proxy for vegetation growth, they can also be misrepresented as snow cover [38], especially during the beginning and end of the seasonal cycle (i.e., outside the growing season), which might introduce uncertainty into the extraction of phenological events [39]. Therefore, we used daily temperature data to identify observations that may have been covered by snow during the non-growing season as those with a temperature below zero for at least five consecutive days and replaced them with the temporally nearest snow-free pixels. This ensured that the extracted phenological events would not extend beyond the thermal growing season and restrict the period of the NDVI analysis.

To extract the phenology from the NDVI time series, we used the Polynomial Curve Fitting method (Polyfit-Mr) from Piao et al. [40] that has been shown to be reliable in regional and global phonological studies [4, 40] (for details, see S2 Fig with the mean annual NDVI data from the grassland as an example). With the Polyfit-Mr, we smoothed the 15-days NDVI composites and interpolated them to a daily resolution using a six-degree polynomial function. Following Liu et al. [41], we optimized the coefficients of the polynomial function using the Levenberg-Marquardt (L-M) method. Then, SOS and EOS could be inferred from the changing characteristics of the seasonal cycle of NDVI over time. The dates with a maximum and minimum change of NDVI were defined as the start (SOS) and end (EOS) of the growing season, respectively. Subsequently, the corresponding NDVI thresholds were used to determine SOS and EOS from NDVI data of each individual year from 1982 to 2011. For a given year, the length of the growing season (GSL) was defined as the time interval between the SOS and EOS. We excluded non-vegetation regions with a mean annual NDVI of less than 0.1 because these lack a visible seasonal cycle according to Zhou’s study [42].

The variation of SOS, EOS, and GSL during the period 1982 to 2011 was estimated using a temporal linear least-squares regression at the pixel level. Following a previously described protocol [43], we resampled the MODIS land-cover map into the same spatial resolution as the phenology data (i.e., 8 by 8 km) using a majority filter (i.e., the predominant land-cover type within a phenology pixel was assigned to this pixel). In addition, we explored the interplay between phenology and the changing climate. To do so, we resampled the satellite-derived SOS and EOS into 0.5° x 0.5° grids. Second, we correlated SOS and EOS with the climatic variables in the month prior to the date of SOS and EOS and summarized the climatic variables for the preseason periods (i.e., preseason) during which the highest correlation was attained. Then, we applied partial correlation analysis among the preseason climate variables, SOS, and EOS. In that way, we were able to quantify the statistical relationship among a single climatic variable (e.g., temperature), SOS, and EOS.

## Results

### Phenological indicators and their temporal trends

**Fig 2:**
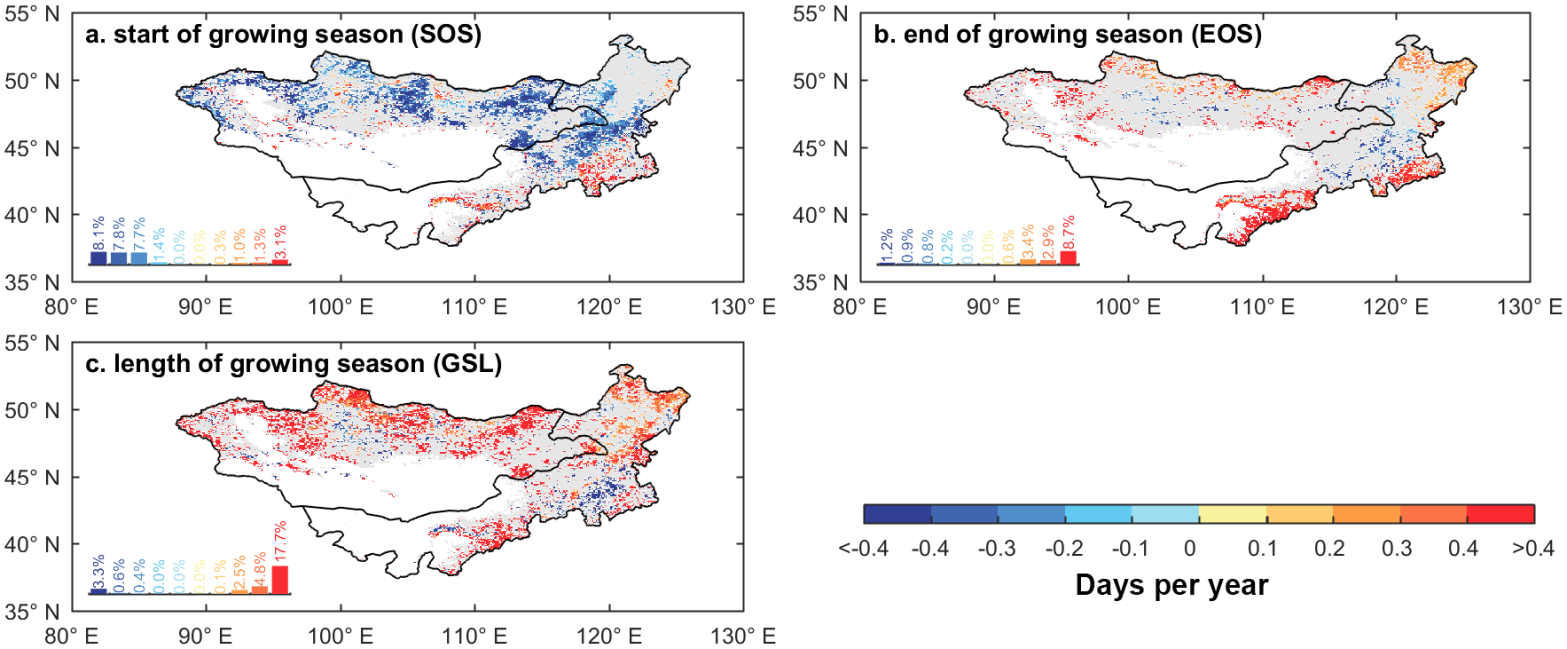
Spatial pattern of linear trends of SOS, EOS, and GSL across the Mongolian Plateau from 1982 to 2011 for a) SOS, b) EOS, and c) GSL. Trends that are statistically significant at p < 0.05 are colored while gray areas indicate regions with insignificant changes. The histograms below each map summarize the distribution of the changes in the maps.

Fig 2 presents the spatial pattern of linear trends in SOS, EOS, and GSL. Fig 2a shows that approximately 25% of the vegetated area (i.e., areas with an NDVI larger than 0.1) on the Mongolian Plateau region (mainly in the Northern and Eastern parts) experienced significantly earlier SOS (*p* < 0.05). On average, the SOS advanced by 0.10 days yr^-1^ from 1982 to 2011. Meanwhile, 16% of the study area experienced a significantly delayed EOS (Fig 2b). Changes in EOS were more spatially heterogeneous than SOS. The average delay of the EOS was 0.11 days yr^-1^, which was slightly larger than the advancing SOS. As a result, the GSL expanded by 0.2 days yr^-1^ on average for the study period. Approximately 25% of the study area showed a significant expansion of GSL (Fig 2c). S3 Fig shows the spatial pattern of the average SOS, EOS, and GSL from 1982 to 2011. The variation in average SOS was between 60 to 180 Julian days and 250 to 270 Julian days for EOS. The GSL was, on average, less than 160 days. As expected, the growing season started much earlier in the southern part of the Mongolian Plateau than in the Northern part.

### Phenological variations by land-cover categories

SOS, EOS, and GSL also distinctly varied among different land-cover categories. As shown in Table 1, significant earlier SOS was observed for 22% of the grassland, with spatial clusters mainly in the western and southern part of the study area. The forest SOS was earlier by 0.06±0.10 days yr^-1^ while for shrubland SOS, no significant changes were found. The changes in EOS and GSL were consistently observed across the three land-cover categories. Grassland, shrubland, and forest displayed significant delays on 29%, 43%, and 34% of their area with average delays of 0.06±0.22, 0.03±0.34, and 0.16±0.09 days yr^-1^, respectively. The earlier SOS (except for shrubland) and later EOS contributed to longer GSL across all land-cover categories (grassland, shrubland, and forest) with 0.17±0.29, 0.03±0.73, and 0.22±0.14 extra days yr^-1^, respectively, between 1982 and 2011.

**Table 1:** Significant linear trends (p < 0.05) of phenological indicators for different land-cover categories from 1982 to 2011.

Phenological indicators	Vegetation type	Mean slope	Std. dev.	Positive (%)	Negative (%)
SOS	Grassland	-0.11	0.20	6.1	22.4
	Shrubland	0.001	0.50	14.0	0.0
	Forest	-0.06	0.10	0.0	0.0
EOS	Grassland	0.06	0.22	29.2	8.5
	Shrubland	0.03	0.34	43.3	0.0
	Forest	0.16	0.09	34.4	0.0
GSL	Grassland	0.17	0.29	34.8	0.0
	Shrubland	0.03	0.73	14.5	0.0
	Forest	0.22	0.14	22.9	0.0

### Climatic determinants of phenological variations

**Fig 3:**
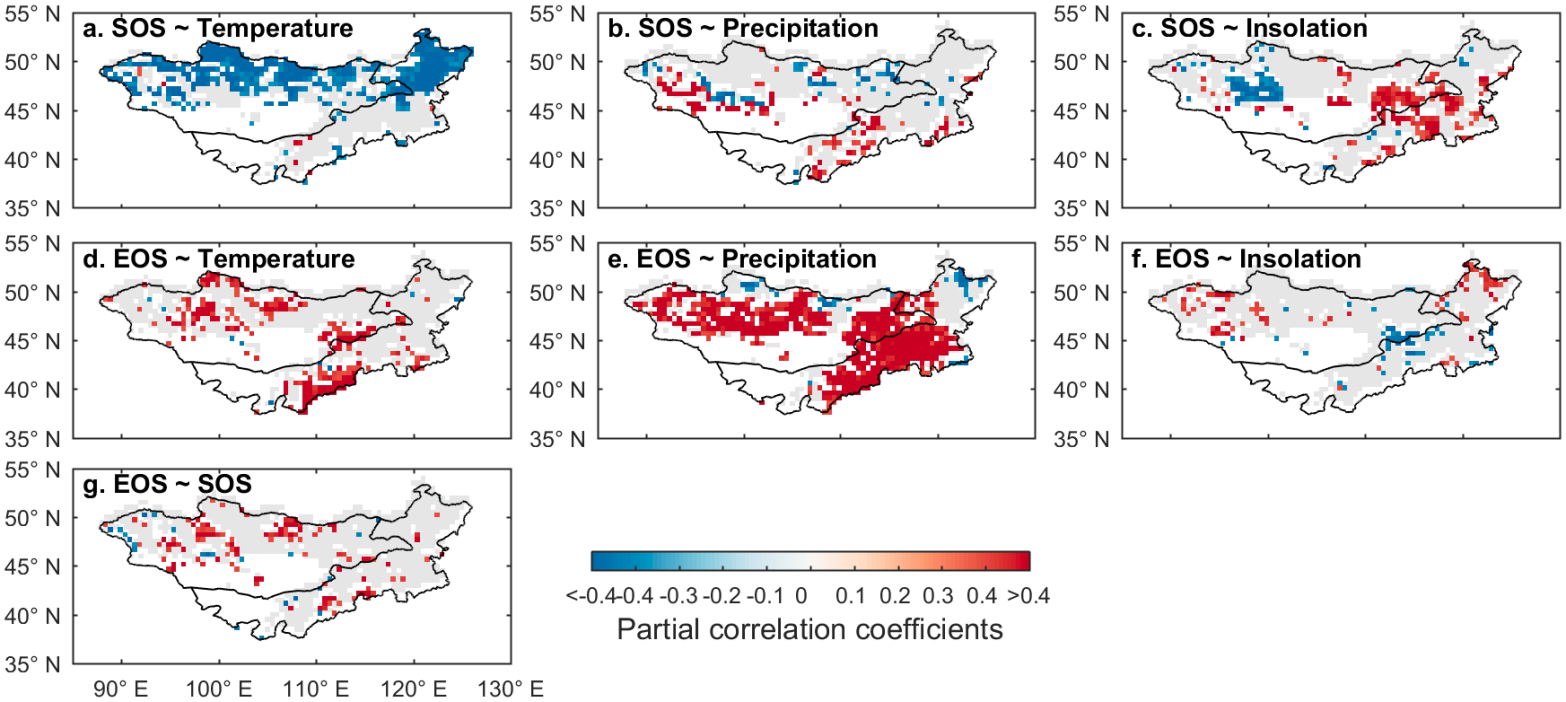
Partial correlation between the phenological indicators (SOS, EOS) and climatic factors (temperature, precipitation, and insolation). Correlations significant at p < 0.05 are in color, and gray areas indicate insignificant relationships between phenology and climatic variables. a: SOS and temperature; b: SOS and precipitation; c: SOS and insolation; d: EOS and temperature; e: EOS and precipitation, f: EOS and insolation, g: EOS and SOS.

To explore the impact of climatic variations on phenological changes, we conducted partial correlation analysis between SOS and EOS with temperature, precipitation, and insolation. The climate factors from the preceding one, two, three, and four months showed the highest correlation with SOS and EOS, with an average of two months (S4 Fig). Significant negative correlations between temperature and SOS prevailed in more than 50% of vegetation area of the Mongolian Plateau, with spatial clusters in northern Mongolia and the northeastern part of Inner Mongolia, indicating that the warming preseason temperatures led to earlier SOS (Fig 3a). The spatial associations of the partial correlations were less uniform for precipitation and insolation (Fig 3b and 3c) with 22% and 26% of study area showing a significant positive correlation, respectively. Temperature tended to postpone EOS (Fig 3d) but precipitation exerted the strongest impacts on the variation of EOS, with approximately 66% of the area exhibiting significantly positive correlations with EOS (Fig 3e). EOS and insolation (Fig 3f) showed only weak and scattered correlations, suggesting that insolation was much less important than precipitation and temperature for determining the changes in EOS. Finally, EOS and SOS were correlated in 18% of the Mongolian Plateau (Fig 3g), particularly in northern Mongolia and some central parts of Inner Mongolia. This suggests that advances in SOS may have resulted in advances in EOS in some areas, though the correlation is much weaker for temperature and insolation.

The partial correlation coefficients between the phenological events (EOS and SOS) and the climatic determinants are summarized in Table 2 for the three land-cover types. In terms of SOS, approximately 39% of the grassland was negatively correlated with temperature (*p* < 0.05). The influence of precipitation and insolation was much weaker, indicating that increasing temperatures may be the main trigger for the earlier start of vegetation growth on the grasslands. Similar patterns were observed for forested areas where the SOS was predominantly negatively correlated with temperature, implying that earlier SOS tended to be associated with higher temperature. The relationship between SOS on the shrubland and climatic factors was less clear-cut, except that precipitation affected the change of SOS with significant and positive correlations in 22% of the shrubland. EOS interacted with climate change in a more complex way: For example, 25% of the observations of EOS on grassland were positively correlated with temperature and precipitation, suggesting that a warming climate coupled with sufficient precipitation before the EOS would facilitate the extension of the growing season in autumn. Shrubland EOS was mainly influenced by precipitation; 62% expressed significant positive correlations. Finally, EOS in forested areas was positively correlated with temperature in 14% and insolation in 27% of its area, while EOS in forests was negatively correlated with precipitation in 26% of its area.

**Table 2:** Percentage of observations with a significant correlation (p < 0.05) between phenological events and climatic variables among different land-cover categories.

Phenological event	Land-cover category	Climate variable	Positive (%)	Negative (%)
SOS	Grassland	Temperature	0.3	39.1
		Precipitation	8.7	6.3
		Insolation	19.1	7.8
	Shrubland	Temperature	4.1	21.6
		Precipitation	23.7	4.1
		Insolation	7.2	5.1
	Forest	Temperature	0.0	94.8
		Precipitation	5.2	1.3
		Insolation	6.5	0.0
EOS	Grassland	Temperature	24.8	0.5
		Precipitation	61.8	1.8
		Insolation	4.9	5.9
	Shrubland	Temperature	13.4	1.0
		Precipitation	45.4	1.0
		Insolation	7.2	3.1
	Forest	Temperature	14.3	0.0
		Precipitation	3.9	26.0
		Insolation	27.3	1.3

## Discussion

Understanding the variability of vegetation phenology over space and time, as well as its climatic determinants, is crucial for better tracing of the ecosystem changes and for better preparing our food systems for the potential effects of future climate change [44]. We explored the spatial and temporal dynamics of three crucial phenological indicators from 1982 to 2011 across the Mongolian Plateau that covers 2.6 million km^2^ across the Inner Asia. We quantified the start (SOS), end (EOS), and length (GSL) of the growing season with a remotely sensed time series of vegetation indices. Specifically, we used the long-term GIMMS NDVI3g records to derive the phenological indicators and analyzed their response to climate change. The results suggest an overall advancement of SOS by 0.10 days yr^-1^, a delay in EOS by 0.11 days yr^-1^, and a prolongation of GSL by 0.21 days yr^-1^.

Others have suggested an earlier SOS of 0.45 days yr^-1^ from 2002 to 2014 that for Inner Mongolia using the double logistic method [26]. However, our findings compare well to results from Northeastern China where 0.13 days yr^-1^ has been suggested using the asymmetric Gaussian, double logistic, and the adaptive Savitzky-Golay filter [45]. In temperate China, 0.11 to 0.30 days yr^-1^ was suggested [41] and across the entire Northern Hemisphere, 0.18 to 0.38 days yr^-1^ from 1982 to 2011 has been proposed, based on a combination of harmonic analysis of time series-maximum (HANTS-Mr), Polyfit-Mr, double logistic, and piecewise logistic methods [4]. The results from this study suggest a 0.2 days yr-1 increase in GSL across the Mongolian Plateau, which is considerably shorter than the (0.84 days yr-1) that Gong et al. [26] found for Inner Mongolia from 2002 to 2014 and much shorter than the 0.6 to 1.0 days yr^-1^ that were suggested for the Tibetan Plateau by Jin et al. [46]. The magnitude of phenological shifts was still within the range of global phenological variation, which suggests that the overall patterns hold true. The differences in the magnitudes of the phenological metrics are due to the different methods, study regions, or periods.

SOS, EOS, and GSL varied considerably among the major land-cover categories. For example, grassland has faster advancing SOS trends but slower delaying EOS trends than forest areas. However, the increase in GSL was highest in forest, followed by grassland and shrubland. This is important because the Mongolian Plateau is dominated by grassland ecosystems with animal husbandry being a major source of livelihoods in rural areas of the region [47]. The earlier start and longer duration of the growing season on the grassland are favorable for vegetation growth and may be positive for the livestock herders. The higher potential for vegetation growth on the grassland of the region may also be the major cause for the observed increase in grassland biomass across the study region [48]. During the past decade, animal numbers over the Mongolian Plateau have been rapidly increasing and constitute an increasingly important factor that regulates the seasonal dynamics of NDVI [18]. Unfortunately, empirical evidence of how the changes in animal density have impacted the NDVI dynamics is scarce but could provide important insights into the effects of livestock grazing as a key determinant of biomass dynamics on the Mongolian Plateau.

Increasing temperature is the main trigger of the advance in the start of the growing season. More precisely, increasing spring temperature may accelerate the accumulation of growing degree days and alleviate negative impacts of the spring frost [49, 50]. Meanwhile, the end of the growing season is likely delayed due to the increasing preseason temperature (S4 Fig), but this delay has been partly mitigated by higher water stress due to lower precipitation in some regions (Fig 3, S4 Fig). Although the increasing temperature could reduce the speed of chlorophyll degradation [51] and the risk of exposure to damaging autumn frost [52], the shortage of water availability enhances the risk of chlorophyll degradation and plant mortality [53, 54]. Many plants, in turn, respond with an earlier EOS to avoid such unfavorable conditions.

The complex interactions between climatic factors and phenology have resulted in heterogeneous EOS trends across the Mongolian Plateau. For example, the advance of EOS for grasslands in northeastern Inner Mongolia and the central part of Mongolia was mainly triggered by decreased precipitation while the delay of EOS in the remaining regions is delayed by higher temperatures. Previous studies reported high positive correlation between SOS and EOS in forested biomes, suggesting that an earlier EOS is accompanied by an earlier SOS [11, 55]. At the genetic level, positive but weak correlations have been found between SOS and EOS in some tree species (e.g., oak and sycamore) [56, 57]. We found the correlation between SOS and EOS to be much weaker for grasslands on the Mongolian Plateau, but field-level experiments in grasslands are required to corroborate this finding for other grassland biomes and at the genetic level. In summary, this study highlight the importance of the temperature effects on SOS while temperature and precipitation jointly influences EOS across the Mongolia Plateau.

Our exploration of the impact of climate change on plant phenology used partial correlation analysis of the climate patterns before the onset of SOS and EOS and neglected the effect of climate variables outside of this period. We found the strongest correlation of climate with EOS for temporal lags between one and four months with an average of two months, suggesting that temperature and precipitation in late summer and autumn were the main factors regulating EOS. Conversely, Xie et al. [58] noted the important influence of summer temperature and precipitation for the timing of EOS. This again suggests that field experiments are urgently required to improve our understanding of plant phenology and its relationship with climatic variables.

## Conclusions

We presented empirical evidence about phenological dynamics for the entire Mongolian Plateau from 1982 to 2011. The growing season in the study area commenced earlier and ended later over the course of this period and has, as a result, considerably elongated. We proved that climatic variations were key determinants of these changes and that temperature changes have been particularly important. The phenological trends were particularly pronounced on the widespread grasslands of the study region, with important implications for grazing livestock and thus rural livelihoods. We found that the earlier start of the growing season was dominated by higher temperature, while insolation and precipitation were of secondary importance. However, the later ends of the growing season were determined by much more complex interactions among climatic variables. Although the warming climate postponed the end of growing season, the water stress due to decreasing precipitation reversed the extension of the growing season length in some grasslands on the Mongolian Plateau. Such insights will be important if we are to anticipate the phenological changes that may occur under future climate change, particularly in arid and semi-arid conditions that are found across much of the Mongolian Plateau because climate change may have yet more profound consequences in such areas for nature and societies.

## Supporting information

S1 Fig Changes in preseason climate across the Mongolian Plateau over 1982–2011. a-c show the changes in temperature mean, precipitation sum and insolation sum during the preseason prior to the date of SOS. d-f display similar findings but during the preseason prior to the date of EOS. Dotted regions suggests significant changes in preseason climate at p < 0.05.
(TIF)

S2 Fig The Polyfit-Mr method for retrieving the start and end of the growing season from an NDVI time series. Black circles in the figure represent the climatology of annual grassland NDVI from the MP. The black line shows the fitted result using six-degree polynomial function.
(TIF)

S3 Fig Spatial pattern of average SOS, EOS, and GSL across the Mongolian Plateau from 1982 to 2011. (TIF)

S4 Fig Length of preseason periods corresponding to temperature, precipitation and insolation. Climatic factors summarized from the date of SOS (a-c) / EOS (d-f) at one-month step, 0 means the current month of SOS / EOS and 1–5 means preseason starts from 1–5 months before the date of SOS / EOS to the current month.
(TIF)
