# Supplementary figures and images for: Changes in vegetation phenology on the Mongolian Plateau and their climatic determinants
Source: PLoS One. 2017 Dec 21;12(12):e0190313. doi: 10.1371/journal.pone.0190313 (PMC5739490; doi:10.1371/journal.pone.0190313)

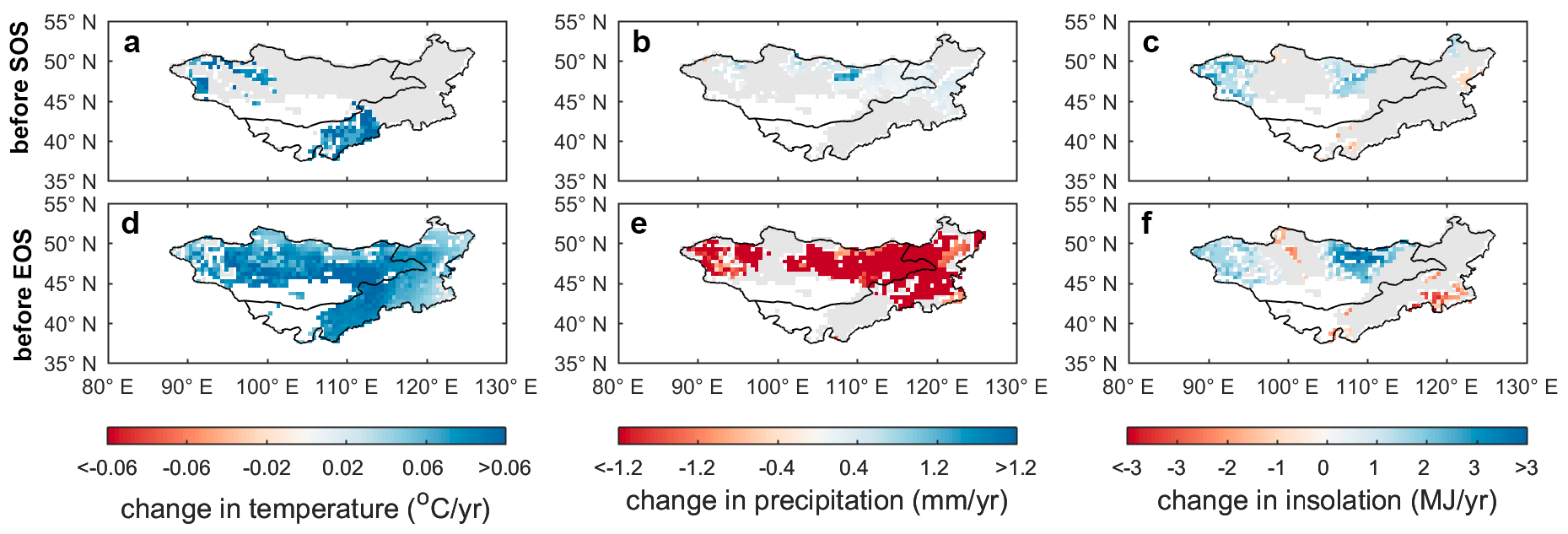

Supplement: S1 Fig — a-c show the changes in temperature mean, precipitation sum and insolation sum during the preseason prior to the date of SOS. d-f display similar findings but during the preseason prior to the date of EOS. Dotted regions suggests significant changes in preseason climate at p < 0.05. (TIF) [file pone.0190313.s001.tif]

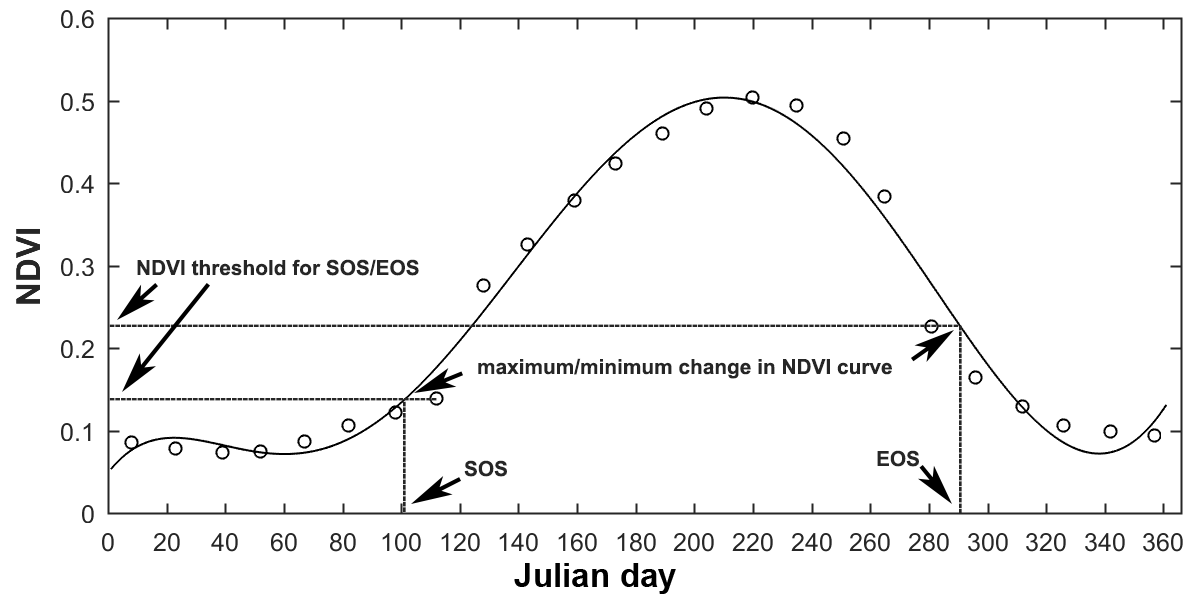

Supplement: S2 Fig — Black circles in the figure represent the climatology of annual grassland NDVI from the MP. The black line shows the fitted result using six-degree polynomial function. (TIF) [file pone.0190313.s002.tif]

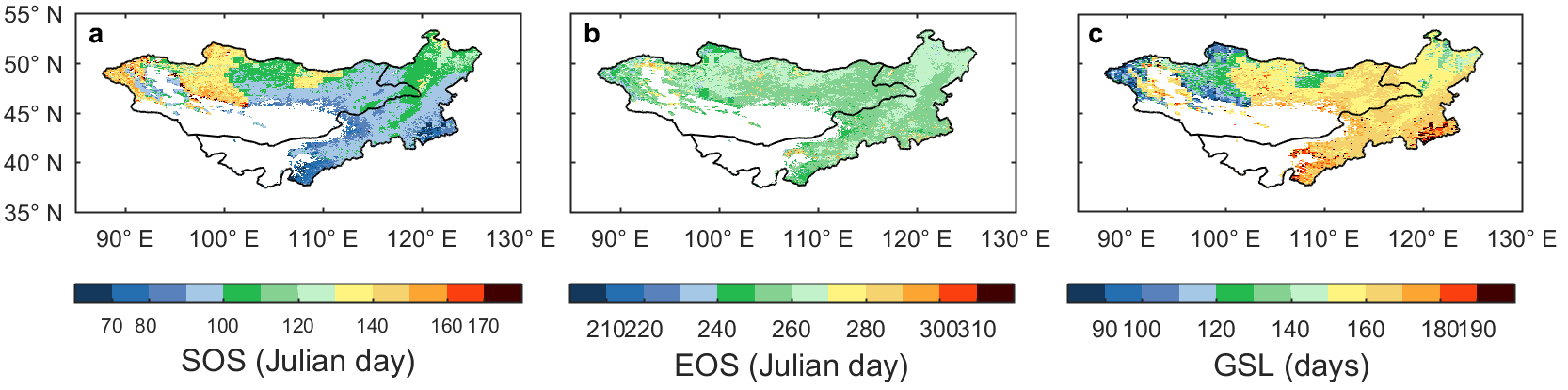

Supplement: S3 Fig — (TIF) [file pone.0190313.s003.tif]

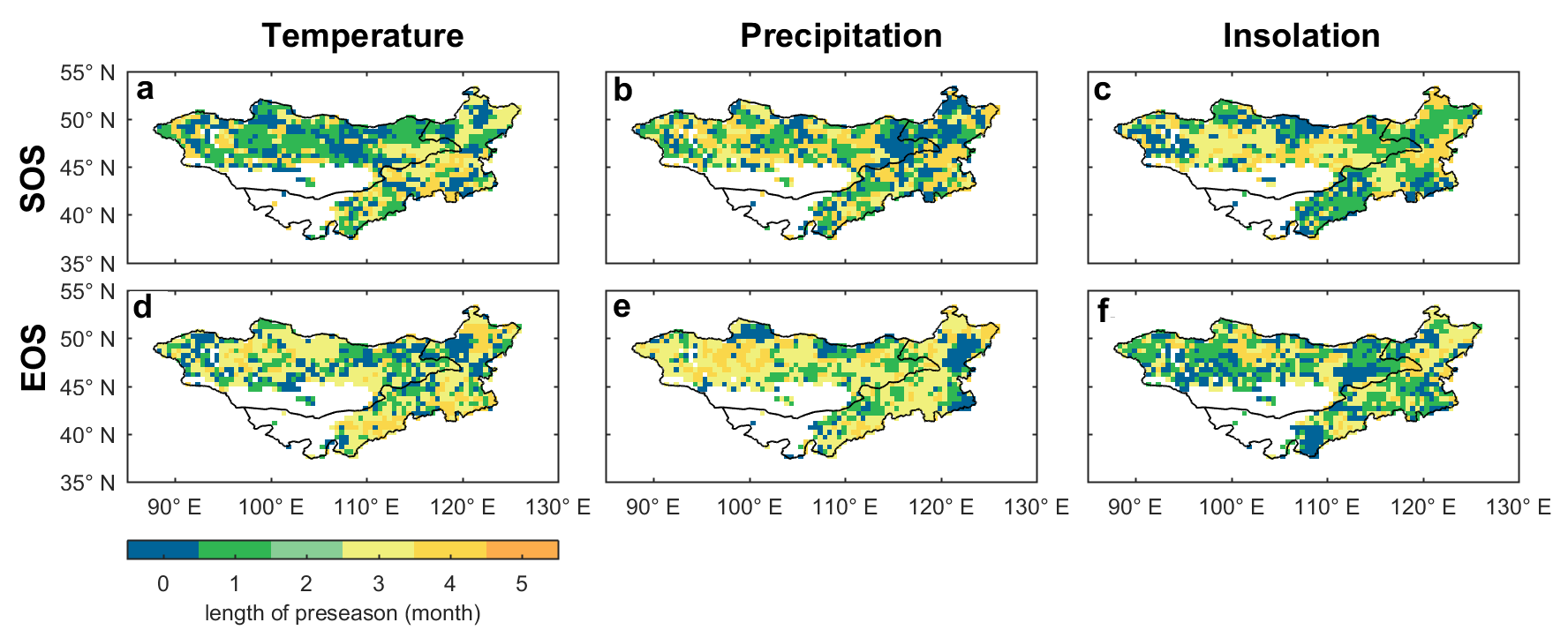

Supplement: S4 Fig — Climatic factors summarized from the date of SOS (a-c) / EOS (d-f) at one-month step, 0 means the current month of SOS / EOS and 1–5 means preseason starts from 1–5 months before the date of SOS / EOS to the current month. (TIF) [file pone.0190313.s004.tif]
